# A Note on the Bacteriological Conditions Present in Ulcers Treated by Oxygen Gas

**Published:** 1897-05-08

**Authors:** D. Semple

**Affiliations:** Surgeon-Major, Army Medical Staff; Assistant Professor of Bacteriology, Army Medical School, Netley


### A NOTE ON THE BACTERIOLOGICAL CONDITIONS PRESENT IN ULCERS TREATED BY OXYGEN GAS.

By D. Semple, M.D., Surgeon-Major, Army Medical Staff; Assistant Professor of Bacteriology, Army Medical School, Netley.

In a very interesting paper read before the British Medical Association at Carlisle, in July last, and reported in the *British Medical Journal* of October 24th, Dr. George Stoker describes in detail his method of the Surgical Uses of Oxygen Gas, and gives a series of cases of chronic ulcers and burns successfully treated by this method. In the same paper he also gives in conjunction with Mr. George Dineen a short bacteriological account of the cases so treated, and a summary of conclusions. With a view to testing the accuracy of these conclusions, advantage was taken of the opportunity of making cultivations from a series of cases of chronic ulcers undergoing the oxygen treatment at the Royal Victoria Hospital, Netley.

The methods pursued were in the main the same as described by Dr. Stoker in his paper, viz., cultivations were made from the ulcers before the application of oxygen and at intervals during the treatment. In all cases the cultivations were made on agar-agar tubes and incubated at 37 degs. C. for periods varying from 24 to 48 hours. Special notice was taken as to whether the favourable progress of healing corresponded with the disappearance of "rod bacteria" in cases where they were present at the outset, and a plentiful growth of staphylococci.

It is to be noted that Dr. Stoker in his paper mentions that staphylococci are favourable, and "rod bacteria" unfavourable micro-organisms when present in a wound undergoing oxygen treatment. He also mentions that soon after the application of oxygen, and when healing sets in, that it is accompanied with a disappearance of the "rods," and a continued growth or increase in growth of the staphylococci.

No attempt was made to classify these "rod bacteria." The majority were short motile rods with rounded ends, growing well on any ordinary medium, and producing liquefaction in gelatine. Between February 19th and April 3rd seven cases of chronic ulcers, all specific except one, were put under the oxygen treatment. The surfaces of the ulcers were attended to in the manner described by Dr. Stoker in his paper. All the patients were men under the age of thirty, and most of them were in a feeble state of health, emaciated and broken down from the effects of constitutional disease and service in India, and were not good subjects to respond to any treatment. Nothwithstanding the inferiority of the material to work upon, marked improvement took place in all at an early stage, pain was diminished, the discharge became less, "rod bacteria" disappeared, and staphylococci flourished all the time.

Case 1: A specific ulcer on the leg of four months' standing. February 19th, half oxygen and half air applied. Improvement was noted on the third day, and continued until March 13th, when the ulcer had quite healed. In this case all the cultivations showed staphylococcus pyogenes citreus only.

Case 2: Rupial ulcerations on arms and legs of five months' standing. February 19th, half oxygen and half air applied to left arm only (the legs and right arm having other treatment). Improvement was noticed on the second day, and on February 27th all the ulcers on the left arm had healed, while for the most part those on the right arm and legs remained open. First cultivation consisted of staphylococcus pyogenes citreus and short "rods." Second cultivation, taken four days after the first, showed staphylococcus pyogenes citreus only.

Case 3: A specific ulcer on the scalp of 13 months' duration. February 20th, pure oxygen applied. February 25th, half oxygen and half air applied. The first cultivation showed staphylococci only. Healing progressed satisfactorily for the first week, when it came to a standstill. After this it was noticed that a new gummatous ulcer had formed close to the old one, a circumstance which interfered considerably with its healing. On March 5th the cultivation showed a copious growth of short "rods," and only a few staphylococci. The ulcer was then sown with a pure culture of staphylococci pyogenes cit. On March 14th, owing to the predominance of short "rods" in the cultivations, the ulcer was again sown with a pure culture of staphylococci pyog. cit. On March 20th the improvement began, and the cultivations at this time showed a plentiful growth of staphylococci and a few "rods" only. On March 28th improvement was again at a standstill, and the cultivations showed "rods" only. On April 1st improvement again set in, and has continued up to date (April 18th). All the cultivations after April 1st showed staphylococci only. In this case delay in healing was accompanied with the presence of "rods" in the cultivations, and when progress set in, these diminished and at last disappeared.

Case 4: Several syphilitic ulcers on arms, legs, and face of ten months' duration; those on the legs being the worst. Patient is in a very feeble state of health and greatly emaciated. February 28th, half oxygen and half air applied to a large ulcer on right instep. Cultivations showed a mixture of short "rods" and staphylococci. Cultivations from another ulcer on right knee, which is being treated in the ordinary way, show a mixture of short "rods" and staphylococci. March 6th, both sides improved. Cultivations from the instep ulcer show staphylococci only, and from the knee ulcer a mixture of "rods" and staphylococci. March 13th, oxygen applied to the knee ulcer. March 18th, both ulcers improving and the cultivations from both show staphylococci only. Oxygen left off on account of the patient being so weak and irritable that he cannot bear the necessary restraint for the continuous application of the treatment.

Case 5: Three ulcers on shin of four weeks' standing, over a large cicatrix and caused by a fall. March 13th, half oxygen and half air applied. Cultivations show a plentiful growth of "rods" and a few staphylococci. March 16th, ulcers healed. Cultivations made on the 15th shows a plentiful growth of staphylococci and a few "rods" only.

Case 6: Gummatous ulcerations on right shin and instep of four months' duration. March 1st, half oxygen and half air applied. No cultivations made before oxygen. Improvement set in after two days' oxygen, and now (April 18th) the ulcers are almost healed. Cultivations all showed staphylococci only (all were taken after oxygen).

Case 7: Lupus of nose and face of nearly three years' standing. Scraping done two days' before oxygen. March 28th, half oxygen and half air applied. No cultivation made before oxygen. April 3rd, marked improvement in ulcers. Cultivations show a plentiful growth of staphylococci only. April 5th, all ulcers closed.

All the foregoing ulcers, with the exception of Case 5, were of a very chronic character, and had shown great resistance to other methods of treatment. In all improvement was noticed in from two to four days after the application of oxygen gas, and, with the exception of Case 3, the improvement was uninterrupted.

A complete explanation as to how this improvement is brought about is at present difficult to give. Possibly the oxygen acts in one or more of the following ways: (*a*) Diminution of irritation. Any dressing you choose to apply to an open sore causes more irritation than a mixture of oxygen and pure air. (*b*) Direct stimulation without irritation. (*c*) The oxygen may oxidise the toxins produced by micro-organisms in the surface of the ulcer. This may apply more especially to the toxins produced by bacilli when present. (*d*) As stated by Dr. Stoker, the oxygen has possibly a selective power in its action on micro-organisms present in the ulcer, encouraging staphylococci, which then outgrow the bacilli. Some of the foregoing points are at present under investigation. Here, as yet, we have only the practical results of a few cases to go by, all of which are in favour of the oxygen treatment.

